# Role of Microbes in the Development of Alzheimer’s Disease: State of the Art – An International Symposium Presented at the 2017 IAGG Congress in San Francisco

**DOI:** 10.3389/fgene.2018.00362

**Published:** 2018-09-10

**Authors:** Tamàs Fülöp, Ruth F. Itzhaki, Brian J. Balin, Judith Miklossy, Annelise E. Barron

**Affiliations:** ^1^Department of Medicine, Division of Geriatrics, Research Center on Aging, Université de Sherbrooke, Sherbrooke, QC, Canada; ^2^Nuffield Department of Clinical Neurosciences, University of Oxford, Oxford, United Kingdom; ^3^Department of Bio-Medical Sciences, Center for Chronic Disorders of Aging, Philadelphia College of Osteopathic Medicine, Philadelphia, PA, United States; ^4^International Alzheimer Research Centre, Prevention Alzheimer International Foundation, Martigny-Croix, Switzerland; ^5^Department of Bioengineering, Stanford University, Stanford, CA, United States

**Keywords:** Alzheimer’s disease, infections, innate immunity, HSV-1, spirochetes, *Chlamydia pneumoniae*, amyloid beta, LL-37

## Abstract

This article reviews research results and ideas presented at a special symposium at the International Association of Gerontology and Geriatrics (IAGG) Congress held in July 2017 in San Francisco. Five researchers presented their results related to infection and Alzheimer’s disease (AD). Prof. Itzhaki presented her work on the role of viruses, specifically HSV-1, in the pathogenesis of AD. She maintains that although it is true that most people harbor HSV-1 infection, either latent or active, nonetheless aspects of herpes infection can play a role in the pathogenesis of AD, based on extensive experimental evidence from AD brains and infected cell cultures. Dr. Miklossy presented research on the high prevalence of bacterial infections that correlate with AD, specifically spirochete infections, which have been known for a century to be a significant cause of dementia (e.g., in syphilis). She demonstrated how spirochetes drive senile plaque formation, which are in fact biofilms. Prof. Balin then described the involvement of brain tissue infection by the *Chlamydia pneumoniae* bacterium, with its potential to use the innate immune system in its spread, and its initiation of tissue damage characteristic of AD. Prof. Fülöp described the role of AD-associated amyloid beta (Aβ) peptide as an antibacterial, antifungal and antiviral innate immune effector produced in reaction to microorganisms that attack the brain. Prof. Barron put forward the novel hypothesis that, according to her experiments, there is strong sequence-specific binding between the AD-associated Aβ and another ubiquitous and important human innate immune effector, the cathelicidin peptide LL-37. Given this binding, LL-37 expression in the brain will decrease Aβ deposition via formation of non-toxic, soluble Aβ/LL-37 complexes. Therefore, a chronic underexpression of LL-37 could be the factor that simultaneously permits chronic infections in brain tissue and allows for pathological accumulation of Aβ. This first-of-its-kind symposium opened the way for a paradigm shift in studying the pathogenesis of AD, from the “amyloid cascade hypothesis,” which so far has been quite unsuccessful, to a new “infection hypothesis,” or perhaps more broadly, “innate immune system dysregulation hypothesis,” which may well permit and lead to the discovery of new treatments for AD patients.

## Introduction

Since the 1907 publication of Alois Alzheimer’s observations of pathological changes in the brain of a woman in her late fifties, these senile plaques and neurofibrillary tangles have become the hallmarks of what is called today Alzheimer’s disease (AD) (Alzheimer, 1907; Alzheimer et al., 1995; Hanger et al., 2014; Sun et al., 2015). Late onset AD, in particular, is the major cause of dementia, and links neurodegeneration and neuroinflammation (Rogers et al., 1992; McGeer and McGeer, 2013; Bolós et al., 2017). The most prominent early symptoms are recent memory loss and language problems (Castellani et al., 2010). For decades, the most strongly prevailing hypothesis for the mechanism of AD has been the so-called ‘amyloid cascade’ hypothesis, which states that pathologically produced amyloid beta (Aβ) is the main cause of the disease (Beyreuther and Masters, 1991; Hardy and Allsop, 1991; Karran and De Strooper, 2016), to the extent that AD has also been termed a ‘proteopathy’ (a disease caused by pathological proteins). The ‘amyloid cascade’ postulates that Aβ fibrils and plaques accumulate leading to synaptotoxicity and neuronal death (neurodegeneration), causing inflammation because they abnormally stimulate microglia (neuroinflammation). Yet, around 400 clinical trials that were predicated on the amyloid cascade hypothesis have failed over the past 14 years; no new AD drugs were approved in that time (Alzheimer’s Association, 2017). One hundred and eleven years since Dr. Alois Alzheimer’s observations, it seems clear that an alternative approach is needed. A hypothesis that can reliably and fully explain the pathogenesis of AD must be found, one that will lead to discoveries of therapies that will be effective for treatment, or even better, for prevention of AD.

For the first time, a new symposium entitled “*Role of microbes in the development of Alzheimer’s Disease: state of the art*” was held at the International Association of Gerontology and Geriatrics (IAGG) in San Francisco, on the 24th of July 2017. Hundreds of scientists attended the Symposium and questioned speakers after the lectures.

The symposium provided an alternative approach to understanding the etiopathogenesis of AD, namely potential microbial infection and innate immune origins for the disease. AD is a complex disease and microbial infection may not be the sole cause of AD, although the studies discussed herein provide compelling evidence of its (infection) important contributions. This hypothesis has slowly gained popularity since the 1970s, despite ongoing strong denial of the idea that microbial infection might play a role in AD. Yet, there is now growing interest and increasing research being done in this field. A number of laboratories have provided strong evidence of the presence of infectious microbes in the brain, from viruses through bacteria to fungi (Balin et al., 1998, 2008; Miklossy, 2011a,b; Itzhaki, 2016; Miklossy, 2016; Pisa et al., 2016, 2017; Alonso et al., 2017; Carter, 2017). The five presenters at the symposium approached this hypothesis from different aspects, but, considering all the experimental data on the development of AD, all came to the conclusion of a possible infectious/innate immune origin for the disease. This can account for the fact that AD develops over decades before clinical symptoms appear (McManus and Heneka, 2017). Clinically, an ongoing redefinition of AD recognizes this by its evolving, changing preclinical symptoms (McKhann et al., 2011; Reiman et al., 2011; Blennow and Zetterberg, 2018; Ghidoni et al., 2018).

A multi-author editorial article on the involvement of microorganisms in AD by Itzhaki et al. (2016) represented a significant event in the field. Additionally, a new book Miklossy (2017) with the participation of more than 30 authors from around the world working on aspects of this topic, appeared in March from IOS Press, the result of a remarkable collaboration in this field.

## The Role of Herpes Simplex Virus Type 1 (HSV1) in Alzheimer’s Disease (AD)

In the 2017 IAGG symposium held in San Francisco, the first speaker was Professor Ruth Itzhaki from the Universities of Oxford and Manchester, and her talk was entitled, **“The role of herpes simplex virus type 1 (HSV1) in Alzheimer’s disease (AD).”**

Dr. Itzhaki’s laboratory first discovered in 1991 that HSV1 DNA is present in a high proportion of the brains of both AD patients and elderly normal subjects (Jamieson et al., 1991). Subsequently, six other groups detected HSV1 in human brain (see review Wozniak and Itzhaki, 2010). The fact that HSV1 is present in elderly normal people as well as AD patients does not preclude a viral role. Most viruses *infect* far more people than they *affect*: genetic factors can determine who is asymptomatic and who suffers disease. Indeed, Itzhaki et al. (1997) found that HSV1 DNA in the brains of carriers of an apolipoprotein E-𝜀4 (APOE-𝜀4) allele confers a high risk of developing AD. A study by another group (Itabashi et al., 1997) confirmed the HSV1-APOE-𝜀4 association in AD, and work on HSV1-infected APOE-transgenic mice has shown that APOE-𝜀4 animals display a greater viral load, and a greater potential for viral damage (see review Wozniak and Itzhaki, 2010). Significantly, APOE-𝜀4 is a risk also for cold sores (Itzhaki et al., 1997; Koelle et al., 2010), which are usually caused by HSV1 in the peripheral nervous system, suggesting that the damage caused by HSV1 is greater, or that repair is lesser, in APOE-𝜀4 carriers.

Subsequently, the Itzhaki lab detected intrathecal antibodies to HSV in cerebrospinal fluid (CSF) of a high proportion of AD patients and healthy elderly people [NB., anti-HSV antibodies found in CSF are known to be long-lived after herpes simplex encephalitis (HSE)]. This indicated that HSV1 can actively replicate in brain, causing damage both directly and via inflammatory processes (Wozniak et al., 2005). It was proposed that reactivation of HSV infection in brain is possibly recurrent, so that damage accumulates, leading eventually to the gradual development of AD.

The next discoveries revealed direct links between HSV1 damage in cell culture, and the damage seen in AD brain. The Itzhaki lab and several other groups found that Aβ and P-tau (Wozniak et al., 2007; Zambrano et al., 2008) and the relevant enzymes that produce them increase greatly in HSV1-infected cell cultures (Ill-Raga et al., 2011 and see review, Itzhaki, 2016). Further, it was found that Aβ deposition occurs in the brains of HSV1-infected mice. They next investigated the proximity of HSV1 DNA to amyloid plaques in human brain, and found a striking co-localisation (Wozniak et al., 2009): 90% of plaques contained HSV1 DNA, and in AD brains, 72% of the viral DNA was associated with plaques (only 24% in elderly normal brains, perhaps reflecting reduced Aβ production or greater clearance). These findings, taken together with Aβ accumulation after infection, suggest that HSV1 causes the formation of toxic Aβ species and plaques, and support a causal role for HSV1 in AD.

The HSV1-induced increase in Aβ suggests that at least initially, the peptide at low levels might act as part of the innate immune system’s response to HSV1, perhaps protectively as a “bioflocculant,” i.e., binding neurotoxic agents (Robinson and Bishop, 2002), or as an antimicrobial peptide (Wozniak et al., 2005), although, in the latter study, its toxicity precluded accurate assessment of any antiviral activity. However, in view of recent positive findings (Bourgade et al., 2015; Kumar et al., 2016), it seems likely that the extent of Aβ’s antiviral activity is determined by both its preparation method and its aggregation state. In any case, though, Aβ eventually becomes toxic, presumably when overproduced, and when oligomerization occurs.

Another important discovery was that HSV1 infection reduces expression of presynaptic proteins synapsin-1 and synaptophysin and decreases synaptic transmission; these inhibitory effects on synaptic function were dependent on GSK-3 activation and subsequent intraneuronal accumulation of Aβ (Piacentini et al., 2015).

Now, following almost three decades of disregard of, or opposition to, the role of HSV1 in AD, there are well over 130 publications using diverse approaches—genetic, immunological and virological—that support this concept, as well as Dr. Itzhaki’s proposal that AD could be treated with antiviral agents (see below).

Reactivation of HSV1 in brain is an essential part of the concept that the virus is a major risk for AD. However, reactivation of virus in HSV1-infected mice was previously found to be much rarer in brain than in the trigeminal ganglia (TG) and was considered to be rare also in humans. Nonetheless, many case studies have provided evidence of “mild” and repeated reactivation in humans (Klapper et al., 1984), recovery from which was almost complete. In fact, there are probably many instances where mild reactivation occurs, but is either undetected, or underdiagnosed, because clinically the person is non-symptomatic.

More direct evidence of HSV1 reactivation in brain was shown in a study of several thousand CSF samples (Peter and Sevall, 2001). The proportion of the samples positive (8 × 10^-3^) for HSV1 was very much higher than expected from HSE prevalence in the population (∼2 × 10^-6^), which is especially surprising as HSV1 DNA persists in CSF for only about a week after HSE (in contrast to long-lived intrathecal HSV1-antibodies), and interestingly, there was a particular bias to females aged over 70 years considering the preponderance of females with AD. These data suggest that HSV1 reactivation in brain might be fairly frequent in the elderly because of their impaired immunity (just as its impairment during aging might lead to entry of HSV1 into the brain). The finding is consistent with an early study which found that HSV DNA was detectable in *post mortem* brain from subjects who had been immunosuppressed and were HSV-seropositive, but not in seronegative nor non-immunosuppressed subjects (Saldanha et al., 1986). Consistently also in mice, Ramakrishna et al. (2015) showed that in HSV1-infected immunodeficient mice, HSV1 is easily reactivated in brain as well as in TG. Yao et al. (2014) examined HSV1-infected mice during latency using a modified *ex vivo* reactivation assay dissociating the CNS explant into single cells. In contrast to earlier results, they found in brain more copies of the viral genome, and also more frequent reactivation, than in the TG. They attributed this to the previous usage of dissociation and mincing of tissue, resulting in greater damage to brain cells than TG cells, with consequent underestimation of reactivation in brain.

Other evidence of reactivation comes from epidemiological investigations of anti-HSV1 IgG and IgM antibodies in serum from AD patients, and measurement of IgG avidity index as an indicator of reactivation. The results show an association between systemic infections and cognitive decline, with HSV1 particularly implicated (see review, Itzhaki, 2016).

Other relevant findings include the following: induction of toll-like receptors in HSV1-infected astrocyte cultures, which has been linked to the likely effects of reactivation of the virus in brain, and dynamic interactions between HSV1 and amyloid precursor protein (APP). APP was found to be present in large numbers, about a thousand or more molecules, in isolated HSV1 particles (Satpute-Krishnan et al., 2003). These virus-APP particles travel together within cells, and in HSV1-infected cells APP distribution is abnormal (Cheng et al., 2011). Nascent HSV1 alters cell membrane organization and anterograde transport, which are essential processes for neuronal function and survival; thus, chronic infection would have a greater impact on these processes (Bearer, 2012).

Genome-wide association studies (GWAS) have linked various AD pathways to those of HSV1 infection (Carter, 2008; Licastro et al., 2015). Although any single gene or SNP effect is very weak (each OR value for AD being less than 2, apart from APOE), a few specific genes, when combined, are strongly associated with AD. These genes might code for proteins that interact in various processes, leading to a synergistic effect on AD development. Environmental factors might trigger certain genes, which then affect other genes, with secondary effects via apoptosis, immune responses, etc. Microbes, especially herpes viruses, were suggested as the possible link for all SNPs with which GWAS have been shown to be associated.

**FIGURE 1:**
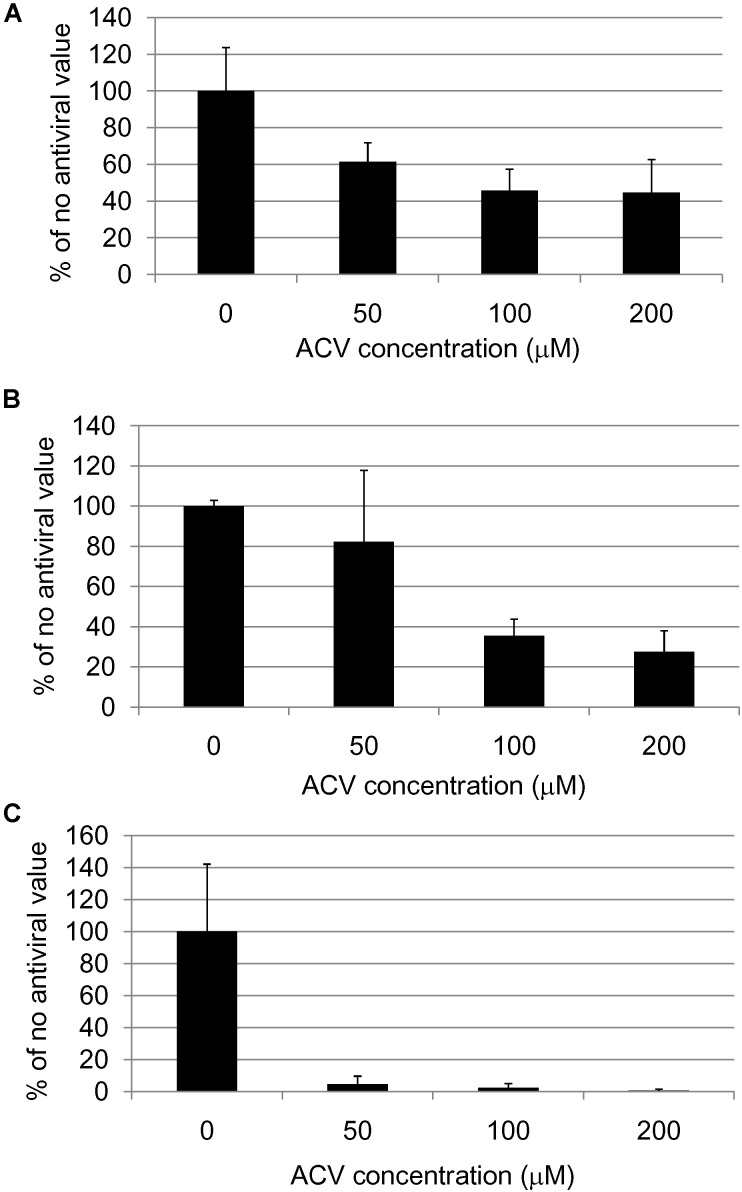
Quantification of **(A)** HSV1 proteins, **(B)** β-amyloid and **(C)** abnormal tau phosphorylation in HSV1-infected cells after acyclovir treatment (Wozniak et al., 2011).

All the above data suggest that antivirals might be used for treating AD (at least in APOE-𝜀4 carriers). Studies on HSV1-infected Vero cells in culture (**Figure 1**) showed that the main anti-HSV antiviral, acyclovir (ACV), inhibits HSV1-induced accumulation of Aβ and P-tau (Wozniak et al., 2011). ACV acts by inhibiting viral DNA replication, so it would inhibit Aβ and P-tau only if their formation depends on viral DNA replication. In fact, ACV and other antivirals did indeed reduce both Aβ and P-tau accumulation (as well as, obviously, HSV1 level); P-tau but not Aβ accumulation was found to depend on HSV1 DNA replication, thus it was directly affected by ACV, while the Aβ decrease was attributed to reduced formation of new viruses, hence reducing viral spread. These results support the treatment of AD by antivirals.

## Alzheimer’s Disease and Spirochetes – a Causal Relationship

The second speaker was Dr. Judith Miklossy, Director of the Prevention Alzheimer International Foundation and International Alzheimer Research Center in Switzerland. Her talk was entitled “**Alzheimer’s disease and spirochetes – A causal relationship.**”

The work presented was a summary of three decades of research based on the generally accepted historical evidence that chronic bacterial infection can cause dementia. Indeed, syphilitic dementia is caused by a peculiar bacterium, a spirochete, called *Treponema pallidum*. In chronic syphilitic infection, particularly in the longstanding atrophic form of general paresis, the most characteristic clinical symptom is dementia and the most characteristic pathology is brain atrophy. Spirochetes accumulate in gray matter areas of the brain, particularly in the cerebral cortex (Merritt et al., 1946; Miklossy, 2008). Hundreds of scientists during the past century have reported and illustrated the accumulation of *Treponema pallidum* colonies in the cerebral cortex (Miklossy, 2015). The formation of cortical spirochetal “masses,” “balls” or “plaques” cannot be distinguished from senile plaques. In a similar way, individual spirochetes cannot be discerned from the pathological filaments called neuropil threads or curly fibers in AD, which diffusely disseminate along the cerebral cortex. Intra-cytoplasmic spirochetes in neurons produce lesions identical to neurofibrillary tangles and granulovacuolar degeneration. The plaque-like structures were frequently in contact with blood vessels, and with the cortical capillary network (Sweeney et al., 2018). These historical data were collected and compared with the characteristic Alzheimer’s-type changes in a recent review (Miklossy, 2015). The results clearly show that *Treponema pallidum* is able to reproduce all the clinical, pathological and biological hallmarks of AD.

Alzheimer’s disease is the most frequent cause of dementia and, following the example of syphilitic dementia, where the cause of dementia is a chronic bacterial infection, the possibility that other types of spirochetes might play a role in the etiology of this devastating neurodegenerative disorder must be considered. Several scientists, from various countries, over the last three decades have undertaken research in this direction. The human body harbors various types of spirochetes. More than 60 different *Treponema* species are found within the oral cavity (Dewhirst et al., 2000; Paster and Dewhirst, 2000), which are present in a large part of the population. Seven of them were shown to be both invasive and predominant periodontal pathogens (Riviere et al., 1991). Six of them were found in the brain in AD (Riviere et al., 2002). These oral spirochetes can reach the brain directly through the olfactory pathway, but also through hematogen dissemination and/or through the lymphatic system. Other spirochetes found in the human body include intestinal spirochetes and various entirely neglected urogenital spirochetes. *Treponema* spirochetes are difficult to cultivate and maintain in pure culture. Still today, *Treponema pallidum* cannot be cultivated and maintained in synthetic medium *in vitro* (Miklossy, 2008). By contrast, another spirochete, *Borrelia burgdorferi*, the etiologic agent of Lyme disease (LD), transmitted by infected ticks and discovered about 30 years ago (Burgdorfer et al., 1982) can be cultivated in BSK or in a modified BSK-II medium, which allowed for more intensive research on the characteristics of this spirochetal infection. Research on Lyme disease gained more attention, because of the increasing number of affected patients worldwide.

MacDonald and Miranda (1987) first reported the presence of *Borrelia burgdorferi* in the brain of a patient suffering from AD in 1987, which was later confirmed by the same and other authors (MacDonald, 1988; Miklossy, 1993; Riviere et al., 2002; Miklossy et al., 2004; Meer-Scherrer et al., 2006; Allen et al., 2016). However, all those authors who investigated the role of *Borrelia burgdorferi* alone in AD, failed to show an association between Lyme disease and AD (Gutacker et al., 1998; Marques et al., 2000; Galbussera et al., 2008; O’Day and Catalano, 2014). The lack of correlation between the incidence of Lyme disease and deaths due to AD cannot reflect the lack of involvement of *Borrelia burgdorferi* in Alzheimer’s dementia (Refer to Letters to the Editor, the 13th of August 2014, Journal of Alzheimer’s Disease)^1^. AD caused by other spirochetes that are more prevalent in the human body (Miklossy, 2011b) can overlap with the small proportion of AD cases caused by *Borrelia burgdorferi* alone. Additionally, in the study by O’Day and Catalano (2014), evidence was not provided on whether the analyzed AD patients had a positive serology for LD or not, whether the LD patients had dementia or not, or whether those that died due to AD were neuropathologically confirmed to have suffered from definite AD. It seems evident that investigating the role of *Borrelia burgdorferi* in Alzheimer’s patients who have no Lyme disease will never succeed. In a similar way, despite the generally accepted knowledge that *Treponema pallidum* can cause dementia, studying thousands of patients with dementia without syphilitic infection would never show the involvement of this spirochete in dementia. Future research on the role of spirochetes in AD should always include various types of spirochetes, which are involved in the disease. The use of negative controls without any Alzheimer’s type changes is also primordial as the process of the longstanding spirochetal infection starts years, frequently decades, before the diagnosis of dementia is made.

Those who have considered that various types of spirochetes may be involved in AD have observed spirochetes in the brains of all Alzheimer’s cases analyzed. Spirochetes were also present in cases with fewer Alzheimer’s type changes (Miklossy et al., 1996; Miklossy, 1998), but their number was lower. In these studies, all control cases used were without dementia and without any Alzheimer’s type changes. When analyzed, these controls were all free of spirochetes (Miklossy, 1993, 1998; Miklossy et al., 1995, 1996). In these studies, detecting all types of spirochetes using neutral techniques, in 114 brains, 83 Alzheimer’s brains, and 31 control brains, a total number of 680 brain and blood samples were analyzed. In AD, more than 91.1% (451/495) of the samples analyzed were positive, while strikingly, the 185 control samples were all negative (Miklossy, 2011a).

The statistical analysis of all data related to the detection of spirochetes in the brain in AD, including all positive and negative results, further confirmed a strong statistically significant association and a high-risk factor between spirochetes and AD. In the 214 Alzheimer’s brains analyzed for all types of spirochetes, 102 of 143 showed the presence of spirochetes and only six out of 71 of the controls were positive (AD and mild AD and controls, *N* = 214, AD 102/143, CTRL 6/71, *P* = 1.5 × 10^-19^, OR = 26, 95% CI = 10–80 Miklossy, 2011a). Whether this strong association between spirochetes and AD satisfies Hill’s criteria for causality was also analyzed. All nine criteria of Hill’s were fulfilled indicating a causal relationship between spirochetes and AD. Thus, prompt action is needed to support this line of research (Miklossy, 2011a).

Besides using numerous techniques, such as *in situ* hybridization, polymerase chain reaction (PCR), and cultivation of spirochetes from affected brain tissue, in order to detect spirochetes in the brain, several other investigations were also performed, which are discussed below. In order to show that spirochetes reproduce the characteristic pathological and biological features of AD, primary cell and organotypic cell cultures were infected by spirochetes (Miklossy et al., 2006). In infected cultures, structures similar to senile plaques, neurofibrillary tangles, and neuropil threads and even granulovacuolar degeneration were all observed, which were not found in uninfected control cultures. Amyloid β precursor protein (APP), beta amyloid and phosphorylated tau were all induced by spirochetes in the infected cultures (Miklossy et al., 2006). These results further demonstrated the causal relationship between spirochetal infection and AD.

It has long been known that spirochetes form clumps or micro-colonies *in vitro* and *in vivo* (Steiner, 1940). Cortical spirochetal colonies in syphilitic dementia were considered as reproductive centers for spirochetes (Steiner, 1940). Spirochetes, including *Borrelia burgdorferi* are able to form biofilms *in vitro* (Sapi et al., 2012). Senile plaques are also reported to contain elements of biofilm constituents (Allen et al., 2016).

Previous work has shown that senile plaques in AD can be produced by spirochetes (Miklossy, 1993; Miklossy et al., 2006). It was shown that APP and/or an APP-like amyloidogenic protein is an integral part of spirochetes indicating that bacterial amyloid contributes to senile plaque formation (Miklossy, 1993). It was anticipated that APP and Aβ, the main components of senile plaques, would also occur in pure spirochetal biofilms, and that bacterial DNA and bacterial amyloid are important components of biofilms in senile plaques.

**FIGURE 2:**
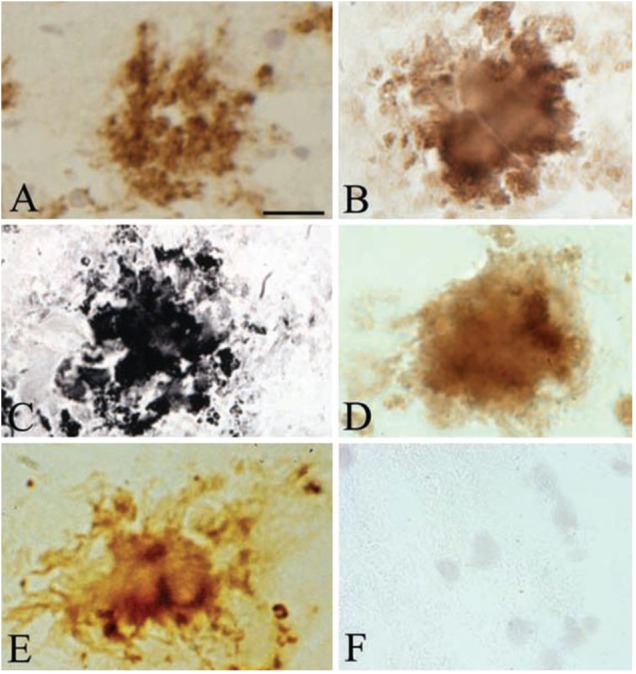
Senile plaques contain spirochete-specific DNA. Photomicrographs of spirochetal colonies or biofilms in an AD case with confirmed Lyme neuroborreliosis where *B. burgdorferi* spirochetes were cultivated from the brain. **(A)** Positive Aβ immunoreaction of senile plaque. **(B)** Senile plaque of the same AD case, as in **(A)**, exhibiting strong immunoreaction for bacterial peptidoglycan. **(C,D)** Photomicrographs showing *B. burgdorferi* antigens in senile plaques immunostained with a polyclonal anti-*B. burgdorferi* antibody **(C)** and with a monoclonal anti-OspA antibody **(D)**. **(E)** *B. burgdorferi* specific DNA detected by *in situ* hybridization in senile plaque of an AD patient where ADB1 strain was cultivated. **(F)** Cortical section of a control case immunostained with a monoclonal anti-OspA antibody showing no immunoreaction. Scale bar **A–E**: 40 μm, **F**: 25 μm. Reprinted from Miklossy (2016), with permission from IOS Press.

Histochemical, immunohistochemical and *in situ* hybridization techniques demonstrated that Aβ and DNA are indeed key components of both pure spirochetal biofilms and senile plaques and further confirmed the biofilm nature of senile plaques (**Figure 2**) (Miklossy, 2016). Extracellularly located DNA fragmentation in senile plaques demonstrated by the TUNEL assay further confirmed that senile plaques are made up by spirochetes and provided biochemical evidence for spirochetal cell death (Miklossy, 2016). Spirochetes evade host defenses, locate extra- and intracellularly, and establish more resistant atypical forms, most notably biofilms, which sustain chronic infection and inflammation and explain the slowly progressive course of dementia in AD.

A review on the involvement of various pathogens related to AD refers to the works of pioneering researchers who have shown the involvement of *Chlamydophila (Chlamydia pneumoniae), Porphyromonas gingivalis* and various other microbes in AD (Jamieson et al., 1991; Kornhuber, 1996; Balin et al., 1998, 2008; Itzhaki and Wozniak, 2008; Miklossy, 2011a,b). Consideration that multi-species biofilms may result in a higher resistance to treatments and a more severe dementia is therefore important. It is noteworthy that co-infecting bacteria, viruses and even fungi frequently occurred in syphilis as well (Miklossy, 2011a).

Other scientists who have participated in this IAGG symposium and presented their three decades of continuous work, along with other more recent observations that Aβ peptide can function as an antimicrobial and antiviral peptide to inactivate invading pathogens and thus is part of the innate immune system, demonstrate that Aβ is not the *cause* of AD but a consequence of *a defense reaction* against infection. Thus, it is critical to include an infectious origin of AD in our current view, and to emphasize future research in this direction.

## Thinking Outside the Box in Alzheimer’s Disease: Could Infection Be the Answer?

The third speaker was Professor Brian Balin from the Center for Chronic Disorders of Aging at the Philadelphia College of Osteopathic Medicine, and his talk was entitled “**Thinking outside the box in Alzheimer’s Disease: Could infection be the answer?**”

Dr. Balin’s talk focused on how infection with the bacterium *Chlamydia pneumoniae* could be a trigger for late-onset AD. In 1998, the Balin group first published on the association of *Chlamydia pneumoniae* in AD brain tissues (Balin et al., 1998). In this report, his team identified this organism in 17 of 19 AD brain tissues using a variety of techniques including: immunohistochemistry, immunoelectron microscopy, electron microscopy, PCR, RT-PCR and *in vitro* cell culturing. Only 1 of 19 control non-AD brains was positive using the same techniques. In evaluating the human brains and the localization of the infection, brain areas that were found to be infected included those connected to olfaction such as the amygdala and entorhinal cortex, as well as the hippocampus proper, and temporal and frontal cortices. All cell types including glia (microglia and astroglia), neurons, and endothelia were infected to some extent. Entry of the organism into the human brain is thought to occur from two potential routes following exposure in the respiratory tract; the first entails following intracellular infection of the olfactory neuroepithelia in the upper nasal airway and then infection traveling to the olfactory bulbs and then deeper into brain structures, and the second route would be following uptake in the lung by monocytes, which survey for infection in lung tissue. The monocytes traffic the organism back into the vasculature from where they can enter the brain through the blood–brain barrier.

Upon finding infection in the AD brains and evaluating by reverse transcriptase PCR, transcripts for *Chlamydia pneumoniae* were obtained from frozen tissue samples. Given these results, it may be possible for some organisms found in frozen tissue to be cultured *in vitro*. To determine if this was possible, homogenates of human brain tissues were cultured with human THP1 monocytes with positive results (Balin et al., 1998). The organism was confirmed to be present and preserved in frozen brain tissues by this method. That the organism propagated *in vitro* intracellularly in the monocytes was confirmed using antibodies specific to *Chlamydia pneumoniae* and by PCR. Propagation in this manner was required as this bacterial organism is an obligate intracellular pathogen that will only grow in an intracellular environment.

The general workflow that Dr. Balin’s lab used in the discovery process was presented to highlight the different steps required to prove how infection is being correlated with the pathogenesis of AD. In this regard, following the identification of *Chlamydia pneumoniae* in the AD brain, animal models (rodents) and cell culture models have been adopted to determine how the organism enters the brain through olfaction, and how cells respond to the organism. Microscopic and culture techniques are routinely used to evaluate infection as it relates to where in the cell the infection resides and how different pathways such as those involving autophagy, apoptosis and inflammasome activation are being activated. Protein (ELISA) and nucleic acid (PCR, RT-PCR) analyses are used to correlate the infection to specific cellular molecular changes.

**FIGURE 3:**
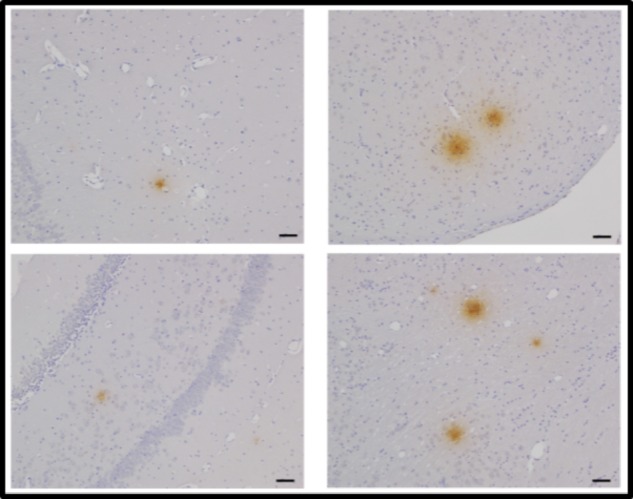
Aβ42 deposits in the CNS at 2 months post-infection following intranasal infection with *Chlamydia pneumoniae*. Brains were examined by light microscopy for the presence of Aβ42 using a specific anti-Aβ 1–42 antibody. Mag bars = 20 μm (Little et al., 2014).

Upon introduction of isolated *Chlamydia pneumoniae* into the nares (or nostrils) of normal non-transgenic laboratory strains of mice (BALB/c or C57BL/6), AD-like amyloid plaques, which labeled with Aβ amyloid 1–42 antibodies, were evident in the brain within 1–3 months post-inoculation (**Figure 3**) (Little et al., 2004, 2014). Aβ amyloid 1–42 immunoreactive plaques were present in peri-rhinal and hippocampal regions. Evidence of the organism in the olfactory neuroepithelia and olfactory bulbs of the animals was obtained by immunohistochemistry and electron microscopy, respectively. Additionally, astrocyte activation was evident following immunolabeling of brain sections with glial fibrillary acidic protein. These studies demonstrated that infection with *Chlamydia pneumoniae* may involve two pathways, the direct olfactory neuroepithelia – olfactory bulbs – entorhinal cortex pathway, and the systemic inhalation – lung – monocyte infection – blood-borne pathway. Interestingly, Dr. Balin pointed out that studies support olfactory impairment with early cognitive change and cited one study by Devanand et al., 2000 in which these authors state “the findings suggest that the inability to recognize smells, combined with a lack of awareness of impaired odor perception, may be a sign of impending Alzheimer’s.”

Additional studies in mice have demonstrated that *Chlamydia pneumoniae* can be recovered from the olfactory bulbs and brain tissues of infected mice following infection up to 4 months post-infection (Little et al., 2005). The recovery is dependent on the infectious dose used for infection with a higher dose 5 × 10^5^ as compared to 5 × 10^4^ IFU being more easily detected. Furthermore, upon consideration of the age of the animals, 20-month-old animals maintained a higher titer of organism after 14 and 28 days post-infection as compared to 6-month-old animals, suggesting that older aged animals did not clear the infection as easily as younger animals. Aging and immunosenescence may contribute to persistent infection. Intriguingly, age is the number one risk factor for AD, but why this is the case has always been questioned. Thus, these data suggest that old animals infected with *Chlamydia pneumoniae* may develop a long-term chronic or persistent infection as compared to young animals.

*In vitro* studies of cellular mechanisms following *Chlamydia pneumoniae* infection are also informative as to the nature of how the organism may traffic through the blood–brain barrier, disrupt pathways such as apoptosis, autophagy and calcium regulation as well as influence the inflammatory response by activating inflammasome complexes. In this regard, infected monocytes placed on a layer of human brain microvascular endothelial cells in a model of the blood–brain barrier transmigrated beyond the barrier to a sub-endothelial chamber (MacIntyre et al., 2002, 2003). The endothelial cells had a transient change in tight junctional proteins as well as increased expression of surface adhesion molecules, ICAM and VCAM. The monocytes increased expression of their integrin surface molecules, LFA1, VLA-4, and MAC-1, suggesting that infection promoted cellular changes that allowed increased interaction of monocytes with brain microvascular endothelial cells to facilitate transmigration through the *in vitro* blood–brain barrier.

*Chlamydia pneumoniae* infection of neuroblastoma cells blocked the apoptotic process (Appelt et al., 2008) as well as activated autophagy and calcium dysregulation. Intriguingly, following infection of monocytes, inflammasome activation was apparent and mRNA and cytokine expression reflecting this inflammatory response was measurable (Lim et al., 2014). All of these features that have been identified with regards to infection with this particular organism previously have been ascribed to AD pathogenesis. A summary of these and a comparison are noted in **Table 1**.

**Table 1:** Comparison between characteristics of Alzheimer’s disease and *C. pneumoniae* infection.

Alzheimer’s disease aspect	*Chlamydia pneumoniae* infection
Olfactory dysfunction	Organism entry
Amyloid deposition	Promotes processing
CNS inflammation	Initiates IL-1β – pro-inflammatory
Cerebral apoptosis	Initiates and inhibits
Mitochondrial dysfunction	ATP sink; ROS target/initiation
Autophagy	Incomplete process
Calcium dysregulation	Increases influx
Neuronal loss	Amyloid fibril deposition
Kynurenine/Quinolinic acid imbalance	Activates IFNγ; shifts to damaging quinolinic acid
Iron dysregulation	Iron sink; deposition
BBB leakage	Increases permeability


Furthermore, links between *Chlamydia pneumoniae* infection and other risk factors for AD are evident. For example, infection is linked to ApoE-𝜀4 expression (Gerard et al., 2005, 2008), atherosclerosis (Filardo et al., 2015), diabetes type 2 (Miklossy and McGeer, 2016), and neurotrauma (as this would affect the blood–brain barrier). These features that correlate with *Chlamydia pneumoniae* infection and AD have led to an infection paradigm, wherein infection with this organism in the brain should be considered a triggering or causative agent in the initiation and perpetuation of AD pathogenesis.

Dr. Balin concluded his presentation with the following quote assigned to Albert Szent Gyorgyi “Discovery consists of seeing what everybody has seen and thinking what nobody has thought.”

## Amyloid Beta Peptides as Antimicrobial Peptides: Relevance for Alzheimer’s Disease?

The fourth speaker was Professor Tamás Fülöp from the Université de Sherbrooke and his talk was entitled “**Amyloid beta peptides as antimicrobial peptides: Relevance for Alzheimer’s disease?**”

He started by stating that if AD is of infectious origin, for which a case was elegantly built by the three first speakers, then perhaps the role of Aβ should be reconsidered. Indeed, some years ago in the field of infection, bacterial amyloid production was considered as antibacterial. Microcin E492 (also called Mcc), for example, is a potent, amyloid-forming, antibacterial bacteriocin produced by *Klebsiella pneumoniae* (Hammer et al., 2008). It can be perhaps speculated that the physiologically produced and circulating Aβ may have such functions. As the microbiota gains importance, it can be hypothesized that amyloid peptides secreted by certain types of bacteria help to maintain the pathological bacteria as well as fungi under control.

The seminal work of the Moir research group shed light on this new concept concerning amyloid peptides as they published data demonstrating both the antibacterial and antifungal properties of Aβ (Soscia et al., 2010). They showed that this peptide is capable of reducing the proliferation of several types of bacteria as well as that of the fungus *Candida albicans*. They hypothesized that Aβ may be a natural antimicrobial peptide (AMP), having an important role in the natural host defense of the organism against different microbes. In this way of thinking they compared Aβ’s effects to another well-known AMP, the cathelicidin peptide LL-37, which is the theme of the last presentation of the symposium by Professor Annelise Barron of Stanford University. Soscia et al. (2010) found that Aβ has somehow more powerful antimicrobial effects than LL-37.

The Dr. Fülöp group became interested in Aβ after this 2010 publication and asked whether this peptide may also have antiviral properties. They started to work on the HSV-1 virus, as it had been shown to be present in AD brain by the first speaker, Professor Ruth Itzhaki. In the meantime, White et al. (2014) published a paper showing that Aβ sufficiently decreased the infectivity of the influenza virus. However, they did not provide details of a convincing mechanism as to how this could happen.

The Dr. Fülöp laboratory set out to demonstrate that Aβ efficiently decreases the intracellular replication of HSV-1 when added before or at the same time as the virus. This seemed to indicate that once the virus is inside of the cells, Aβ has no effect. This led to the hypothesis that Aβ may act extracellularly, which was confirmed by several experiments (Bourgade et al., 2015). Indeed, through confocal microscopy studies, an extracellular interaction was confirmed between HSV-1 and Aβ. Interestingly, influenza virus from the White study and HSV-1 from the Dr. Fülöp study were both enveloped viruses. This led to the hypothesis that Aβ may be effective only against viruses that possess an envelope. In fact, when adenovirus, which does not have an envelope, was used, the Aβ peptide had no effect when added to culture (Bourgade et al., 2015).

**FIGURE 4:**
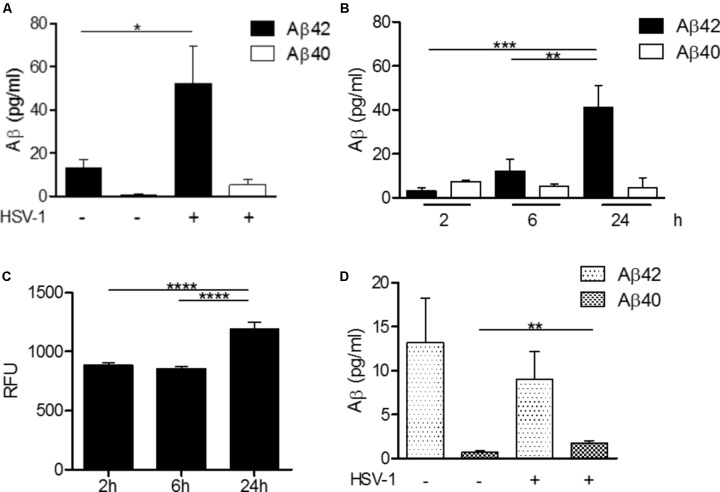
HSV-1-dependent Aβ production in H4 neuroglioma and U118-MG glial cells. **(A)** H4 neuroglioma cells were cultured in the absence (–) or presence (+) of HSV-1 for 24 h and production of Aβ42 and Aβ40 was quantitated by ELISA assays. **(B)** Time-course production of Aβ42 and Aβ40 by H4 neuroglioma cells cultured in the presence of HSV-1. **(C)** Time course of BACE-1 activity in H4 neuroglioma cells exposed to HSV-1 expressed in relative fluorescence units (RFU). **(D)** Aβ42 and Aβ40 production by U118-MG glioblastoma cells exposed to HSV-1 for 24 h, as determined by ELISA assays. Data are shown as the mean ± SEM of four to five experiments performed in duplicate. ^∗^*p* < 0.05, ^∗∗^*p* < 0.01, ^∗∗∗^*p* < 0.001, ^∗∗∗∗^*p* < 0.0001. Reprinted from Bourgade et al. (2016b), with permission from IOS Press.

To be closer to physiological conditions, Dr. Fülöp’s laboratory established an *in vitro* co-culture of microglial cells and neurons. The aim was to study the effect, if any, of HSV-1 infection of neurons on Aβ production (Bourgade et al., 2016b). Indeed, the neurons infected by HSV-1 produced Aβ (**Figure 4**). This confirmed that a viral infection can induce detectable Aβ production. The next step was to study whether β-secretase (BACE) activation is the origin of this increased Aβ production. The experiments showed that BACE was activated in neurons and that inhibition by common BACE inhibitors decreased production of Aβ. Concomitantly, the infectivity of HSV-1 increased as almost no Aβ was produced. Thus, not only was Aβ secretion stimulated by the increased activation of BACE, but also it provided antiviral activity. To confirm that neurons were stimulated by HSV-1, Dr. Fülöp applied the supernatant to neurons before *de novo* HSV-1 infection. Notably, this conditioned media was able to inhibit HSV-1-driven infection of the neurons almost completely. To confirm that this was the effect of secreted Aβ from neurons, Dr. Fülöp neutralized IFNα/β and Aβ, but only the latter resulted in increased infections. So it was not the IFNα/β that acted as an antiviral agent but the Aβ peptide. This again confirmed the specific role of Aβ *in vitro* as a powerful AMP secreted by neurons during acute infection, which renders the neighboring cells resistant to infection. In a microglia/neuron co-culture Dr. Fülöp demonstrated that the microglia phagocytose at least in part the Aβ produced by the neurons, trying to extract a potentially harmful substance like Aβ from the environment. However, it was also found that this phagocytosis process caused the microglia to become activated.

The last part was devoted to better understanding the mechanism of Aβ’s antiviral activities and give a broader sense to this mechanism as well as to the antiviral AMP activity of Aβ (Bourgade et al., 2016a). It was shown that the Aβ and the gB binding protein on the envelope surface of the virus HSV-1 have structure homology which might explain partly its mechanism of action. This interaction occurs in the extracellular space and has two possibilities. The first possibility is a mechanism of binding directly to the binding site of mHSV-1, subsequently inhibiting its entry into the cells. There are some FRET data indicating a very close physical interaction between Aβ and viruses (unpublished data). The second possibility, which does not totally exclude the first, is that Aβ may be a membrane-disruptive peptide or it crosses membranes causing internal damage. There are limited experimental data on artificial membranes showing that this possibility may exist (Arispe et al., 2007). Whatever its exact mechanism of action, the Aβ peptide is able to significantly decrease infectivity of HSV-1 and other viruses, which is highly significant.

Together these data may be integrated into a common hypothesis in which production of Aβ may initially be beneficial at the beginning of an infection, as an AMP helps to contain the original pathogen (Bourgade et al., 2016a; Le Page et al., 2017). In the decades preceding the full clinical manifestation of AD, there may be repeated reactivation of the chronic latent infections, constantly producing new Aβ peptides in response, which cannot be cleared by microglia, progressively leading to neurodegeneration and neuroinflammation. Clinical AD then becomes apparent when the threshold for these pathological changes is exceeded. This vision allows us to also better understand the roles of different risk factors for AD. Furthermore, new targets for treatment could be developed from this approach of AD.

## Evidence that the Human LL-37 May Be a Binding Partner of Aβ and Inhibitor of Fibril Assembly

The last speaker was Professor Annelise Barron from Stanford University, and her talk was entitled “**Evidence that the human LL-37 may be a binding partner of Aβ and inhibitor of fibril assembly.”**

This last talk was relevant to all of the previous four talks, as it was focused on a potential direct binding interaction between the well-known Alzheimer’s-associated Aβ peptide, and the abovementioned, ubiquitous human host defense peptide, the human cathelicidin peptide LL-37, as a possible mechanism that could prevent pathological over-accumulation of Aβ plaques. It connects Aβ accumulation with an inadequate innate immune host defense to infections (given that LL-37 is a critical antimicrobial peptide).

Dr. Barron’s talk was relevant to the concept that infections of brain tissue, whether of viral or bacterial origin, will engender an innate immune response, which in the case of Alzheimer’s patients, may be ineffective or somehow misregulated, resulting in pathological overaccumulation of amyloid, as well as progressive neurodegeneration and neuroinflammation. Indeed, throughout the past 111 years since the first report of AD, the natural physiological function of the Aβ peptide has remained mysterious. Yet recent results of the Moir and Fülöp groups, in just the last decade, now make a convincing argument that Aβ is a key element of human innate immunity, which in addition to being capable of directly inactivating viruses such as HSV-1 (Bourgade et al., 2015, 2016a) and some types of bacteria (Soscia et al., 2010), can also serve to sequester pathogens of either viral or bacterial origin within amyloid plaques, where these pathogens or their remains have been consistently identified, as was discussed by the first three speakers.

Dr. Barron introduced the idea, first proposed in a paper published in July 2017 (the same month that the IAGG symposium was held), that the Aβ peptide and LL-37 peptide may in fact be co-regulated, interacting host defense peptides, which are able to bind to and inactivate each other (De Lorenzi et al., 2017); and that further, if these two peptides are misregulated with regard to their expression, this misregulation might contribute to development of AD.

The identification of physiologically relevant binding partners of Aβ that can modulate or prevent *in vivo* Aβ fibril and plaque formation is important, as this could yield new insights into AD etiology, and moreover may suggest new therapeutic or prevention strategies. The binding partner of Aβ peptide that Dr. Barron and collaborators have identified is the cathelicidin peptide, LL-37, which is an antiviral, antibacterial, antifungal innate immune effector and modulator, ubiquitous in human tissues and expressed in myriad cell types.

Dr. Barron presented the first *in vitro* experimental evidence supporting the hypothesis that LL-37 binds to Aβ peptide and modulates or prevents fibril formation. Her results showed that LL-37 and Aβ bind to each other strongly and form a complex, such that the normal effects of both peptides are ameliorated, i.e., the complex is virtually non-toxic; and also, soluble.

Key collaborators on the project included Dr. Marcella Chiari, Dr. Ersilia De Lorenzi, Dr. Carlo Morasso, and Dr. Renzo Vanna, all from Italy; and Dr. Patrick McGeer from British Columbia. Previous work of Dr. Barron with Dr. McGeer, published in 2015 (Lee et al., 2015) had shown that LL-37 peptide can be detected by Western blots in many different human tissues (indeed, all tissues that were tested), but is most highly expressed in the human gut and brain. Moreover, strong upregulation of LL-37 in a human cadaver with infection (in the lungs of a person who had died of pneumonia) was confirmed; and upregulation of LL-37 in the brain of a patient who died of AD was found. More work needs to be done, with larger numbers of human brains, to verify these findings. In cell culture studies, it was shown that similarly to Aβ peptide, LL-37 peptide can induce neuroinflammation; specifically, it can induce human microglia to release inflammatory cytokines TNF-α and IL-6 (Lee et al., 2015).

Dr. Barron described a series of *in vitro* studies that proved a binding interaction between these host defense peptides, which happen to have almost identical molecular weights (LL-37 is 37 amino acids long, while the Aβ peptide is 40-42 amino acids long), and which are opposite in charge at physiological pH (LL-37 has +6 charge, while Aβ has -3 charge), favoring binding. The two peptides have other sequence complementarities, and further studies of their interactions are being done by Dr. Barron and collaborators (De Lorenzi et al., 2017).

**FIGURE 5:**
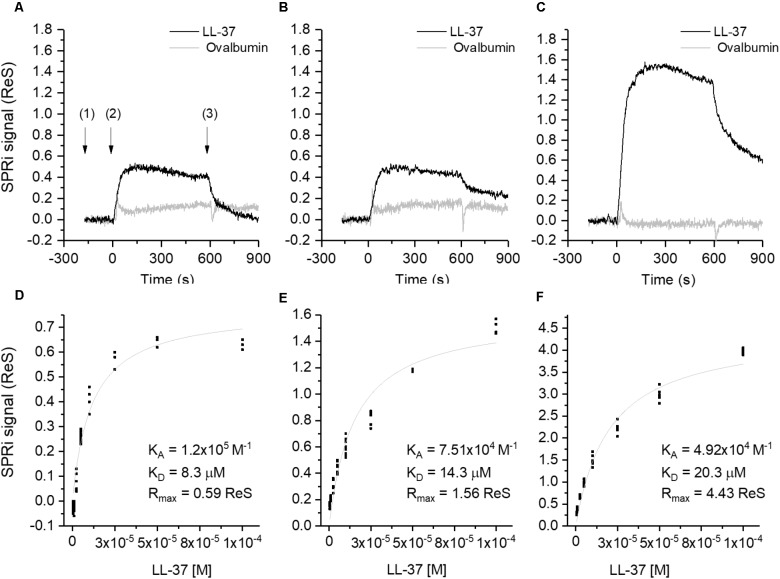
Binding studies performed by SPRi. The SPRi-chip was functionalized with Aβ40 (*t* = 0 days) **(A,D)**; Aβ42 (*t* = 0 days) **(B,E)** and Aβ42 (*t* = 24 days) **(C, F)**. All the Aβ peptides were immobilized in replicate (*n* = 8) on the same SPRi chip at the same concentration (20 μM). SPRi reference-corrected responses related to LL-37 (10 μM) (black) and ovalbumin (10 μM) (gray) (negative control) flowed on the SPRi-chip functionalized with different Aβ forms **(A–C)**. The three SPRi sensograms show the injection of running buffer (baseline) (1), the injection of the analyte (association phase) (2) and the subsequent injection of buffer (dissociation phase) (3). Calibration curve of LL-37 flowed onto different Aβ forms immobilized on the SPRi-chip Aβ40 (*t* = 0 days, **D**) Aβ42 (*t* = 0 days, **E**), Aβ42 (*t* = 24 days, **F**). The equilibrium binding constants (*K*_A_ and *K*_d_) values were calculated using a non-linear curve fit of the SPRi response at equilibrium. Reprinted from De Lorenzi et al. (2017), with permission from IOS Press.

Surface Plasmon Resonance imaging (SPRi) showed strong sequence-specific binding interactions between LL-37 and Aβ (**Figure 5**), with binding being quantitatively assessed with Aβ in different aggregation states (small oligomers, larger oligomers, and mature fibrils, as assessed by capillary electrophoresis analysis). SPRi showed binding specificity between LL-37 and Aβ, that is, Aβ did not bind to an unrelated peptide nor to a scrambled-sequence version of LL-37. The binding constants are of the same order of magnitude expected for specific protein–protein binding interactions. Interestingly, LL-37 peptide binds more *strongly* (as measured by equilibrium dissociation constants, *K*_d_) to Aβ *oligomers* than to mature Aβ *fibrils*, which means that the presence of LL-37 tends to prevent the formation of Aβ fibrils. Indeed, TEM analyses also showed that LL-37 inhibits Aβ42 fibril formation, particularly Aβ’s ability to form long, straight fibrils characteristic of AD. Circular dichroism studies of the two peptides in solution, separately and in equimolar amounts, revealed that the presence of LL-37 can entirely prevent Aβ42 from adopting β-type secondary structure, which is prerequisite to its formation of oligomers and fibrils (De Lorenzi et al., 2017).

Respective cytotoxicities of LL-37 and Aβ42 and their complexes were evaluated in primary human microglia as well as neuroblastoma cell lines (SH-SY5Y), cultured in different experimental conditions (alone or in co-culture). It was found that microglia-mediated toxicities of LL-37 and Aβ42 to neurons in co-cultures (i.e., their tendency to release proinflammatory cytokines) were greatly attenuated when the peptides were co-incubated before addition and allowed to bind. Indeed, the complex of Aβ42 and LL-37 was found to be both soluble, and virtually non-toxic and non-inflammatory (De Lorenzi et al., 2017).

Previous reports indicate that biophysical activities and signaling functions of Aβ and LL-37, which is the only cathelicidin-derived innate immune system peptide found in humans, are connected with each other *in vivo*. For example, the vitamin D receptor (VDR) and retinoid X receptor (RXR) are both relevant to the progression of AD, as well as with expression levels of Aβ and LL-37. Vitamin D3 treatment has been shown to reduce intracerebral amyloid accumulation and to improve cognition in a mouse model of AD (Durk et al., 2014), while RXR activation reduced neurodegeneration and improved cognition in an aggressive mouse model of AD (Mariani et al., 2017). Expression levels of the CAMP gene that encodes hCAP-18, the precursor for LL-37 (Burton and Steel, 2009), are upregulated by activation of the VDR pathway (Gombart et al., 2005), with an obligate involvement of the RXR receptor as well (Cederlund et al., 2014).

Other literature reports suggest that the biophysical activities and signaling functions of Aβ peptides and LL-37 are related *in vivo*. For instance, the Formyl-like Peptide Receptor 1 (FPRL1) is activated by both Aβ (Le et al., 2001) and LL-37 (Yang et al., 2000). This receptor is reportedly involved in inflammatory aspects of AD (Cui et al., 2002) via its effects on phagocyte responses (Iribarren et al., 2005); and a decreased phagocyte clearance of CNS β-amyloid is a hallmark of AD (Mawuenyega et al., 2010). LL-37 itself is involved in monocyte and macrophage autophagy; its expression, stimulated by vitamin D3 (Gombart et al., 2005), engenders enhanced autophagy (Yuk et al., 2009). Thus, a dearth of LL-37 generally will reduce phagocytic activity. Treatments with phenylbutyrate and vitamin D3 have both been shown to be neuroprotective in AD mouse models (Cuadrado-Tejedor et al., 2013; Durk et al., 2014), and these same two compounds are known to be strong inducers of cathelicidin expression (Steinmann et al., 2009). Finally, as discussed above, there is mounting evidence that innate immunity plays a larger role in AD than previously thought, and that Aβ’s normal function may be as an antimicrobial and antiviral peptide (Soscia et al., 2010; Bourgade et al., 2015; Kumar and Moir, 2017). Kumar et al. (2016) showed that Aβ expression protects against fungal and bacterial infections in mouse, nematode, and cell culture models of AD. And, part of the body’s natural response to infection is, of course, to upregulate expression of cathelicidin peptide LL-37, if possible (Burton and Steel, 2009).

To date, most approaches to studying AD rely on the supposition that pathological overexpression or hindered degradation of Aβ lays the foundation for disease. Certainly, overexpression of Aβ, given that it has been proven by the Moir and Fülöp groups to have both antibacterial and antiviral capabilities, is to be expected in the case of tissue response to an infection by pathogens such as HSV-1, spirochetes, or *Chlamydia pneumoniae*, all shown by the other speakers to be relevant to AD.

Dr. Barron raised the novel question here: what if a chronic *underexpression* of another host defense peptide, LL-37, which normally opposes Aβ fibril and plaque formation, plays a key role in the etiopathogenesis of AD? Underexpression of LL-37, which might result from (for instance) insufficient vitamin D3 (i.e., insufficient exposure to sunlight, or intake of fish oil in a northern climate), insufficient dietary retinoids (vitamin A), or other factors, resulting in a low expression of cathelicidin peptide LL-37, would reduce a patient’s ability to fight brain infection. Hence, all of the concepts appear to be connected in a clear way. Dr. Barron and her collaborators have proposed that LL-37 and Aβ42 are natural innate immune binding partners, which implies that their balanced spatiotemporal expression could modulate AD initiation and progression. If spatiotemporally balanced expression of LL-37 and Aβ is not achieved, for instance because of dietary or lifestyle factors, then it would be expected that the Aβ fibrils could pathologically accumulate, as occurs in AD.

If this hypothesis is correct, then rather than ‘pathological overexpression’ of Aβ, chronic *underexpression* of LL-37 (which would also create an ineffective response to infection) is the problem that leads to AD pathology. It seems to be a rather simple explanation. If it is true, how could it be explained that it took 111 years to discover this? It is difficult for researchers to identify a systemic element that *should* be present, or perhaps should be better regulated, but is not. It is natural to study what *is* present, rather than what is *not*.

Finally, Dr. Barron stated that she is now conducting an *in vivo* study of this hypothesis, and has identified, with collaborator Dr. Mehrdad Shamloo at Stanford, an oral polytherapy of compounds that given together can strongly induce the expression of the endogenous cathelicidin (murine cathelicidin—highly similar to LL-37—is called CRAMP). Early results of an extended feeding study using both wild-type and the 5XFAD Alzheimer’s mouse model indicate that these two peptides also interact and can neutralize each other’s biophysical and biological activities *in vivo*, as was found *in vitro*. If Dr. Barron’s hypothesis is correct, stimulation of the key human antimicrobial and antiviral peptide LL-37, together with antiviral drug treatment, might help prevent or treat AD in patients who suffer from viral or bacterial infections of their brain tissue. Early treatment would be key. Additionally, further studies are required to establish the exact role of such associations in AD.

## Conclusion

In summary, the five speakers provided strong evidence supporting the role of chronic infections in the development of AD, as well as a potential misregulation of the human innate immune response to these infections, as a plausible route for new investigations of the etiology and pathophysiology of AD (**Table 2**). Investigations along these lines are warranted, given the inadequacy of the amyloid cascade hypothesis, which still remains the dominant framework for studying AD. This new paradigm may provide novel targets for the prevention and treatment of this devastating disease of the elderly. Much more work is needed in this field of research, but the hope is that many scientists will join this endeavor for the benefit of AD patients around the world; and indeed, to potentially achieve early diagnosis of various infections, viral, fungal and bacterial, which can predispose certain patients to the initiation and progression of AD decades prior to the disastrous clinical manifestations.

**Table 2:** Summary of key findings.

Title	Author	Highlights
The role of herpes simplex virus type 1 (HSV1) in Alzheimer’s disease (AD)	Ruth Itzhaki	• HSV1 DNA resides latently in brain of many elderly and can reactivate; damage is probably limited and localized.
		• HSV1 DNA in brain and ApoE-𝜀4 confer a strong risk of AD; ApoE-𝜀4 is a risk for cold sores.
		• HSV1 infection of human brain cells and of mouse brain causes AD-like changes.
		• HSV1 DNA is located precisely within amyloid plaques in AD brains.
		• Antiviral treatment reduces greatly Aβ and P-tau production.
		• Vaccination of mice prevents HSV1 latency in brain.
		• ApoE determines severity of damage (or susceptibility to infection) by diverse pathogens.
Alzheimer’s disease and spirochetes – A causal relationship	Judith Miklossy	• Chronic bacterial infections can cause dementia (e.g., the spirochete *Treponema pallidum* causing syphilitic dementia).
		• Spirochete colonies in brain can produce plaque-like structures indistinguishable from senile plaques.
		• 451/495 AD blood and brain samples had spirochetes detected compared to none found in 185 controls.
		• APP, Aβ, P-tau induced by spirochetes in infected cultures.
		• Aβ and DNA are key components of pure spirochetal biofilms and senile plaques.
		• Hill’s criteria support a causal relationship between spirochetes and AD.
		• Pathological process begins long before diagnosis of dementia; appropriate antibiotic treatment may result in regression and, if started early, prevention of dementia.
Thinking outside the box in Alzheimer’s disease: Could infection be the answer?	Brian Balin	• *Chlamydia pneumoniae* (Cpn) found in abundance in AD brains (17/19) compared to controls (1/19).
		• Viable Cpn can be isolated from AD brains and propagated in human THP1 monocyte cell cultures.
		• Cpn introduced into nostrils of healthy BALB/c mice results in AD-like amyloid plaques in brain.
		• Infection occurring through direct olfactory neuroepithelia pathway or systemic inhalation pathway.
		• Cpn infection shares similar risk factors and characteristics with AD (**Table 1**).
Amyloid beta peptides as antimicrobial peptides: Relevance for Alzheimer’s disease?	Tamàs Fülöp	• Aβ exhibits antiviral activity against enveloped viruses.
		• Neurons produce Aβ peptides in response to HSV1 infection that is time dependent and requires β-secretase activity.
		• Most likely mechanism of action of Aβ against HSV1 is disruption of viral insertion into external membrane, interfering with its ability to infect host cells.
		• Aβ42 induces activation of microglia in response to reactivation of HSV1.
		• Overproduction of Aβ42 could cause sustained neuroinflammation, which, over a long term, would manifest as AD symptoms.
		• Aβ may be at first beneficial in its response to infection, but later becomes detrimental.
Evidence that the human LL-37 may be a binding partner of Aβ and inhibitor of fibril assembly	Annelise Barron	• Aβ and human innate immune peptide, LL-37, may be co-regulated; misregulation may contribute to AD development.
		• LL-37 is present in many human tissues, most notably brain and gut; strongly upregulated in an AD patient’s brain.
		• SPRi shows strong sequence-specific binding between LL-37 and Aβ; stronger binding to Aβ oligomers than fibrils.
		• TEM demonstrates LL-37 can inhibit Aβ fibril formation.
		• Microglia-mediated toxicities of LL-37 and Aβ to neurons in co-cultures is greatly reduced when peptides are co-incubated prior to addition.
		• Biophysical activities and signaling functions of the two peptides may be linked *in vivo*.
		• Underexpression of LL-37 may lead to accumulation of Aβ and development of AD.

## Author Contributions

RI, JM, BB, TF, and AB have written their respective parts, reviewed, and commented on the entire article.

## Conflict of Interest Statement

TF is a consultant for Eisai. The remaining authors declare that the research was conducted in the absence of any commercial or financial relationships that could be construed as a potential conflict of interest.

## Footnotes

1 https://www.j-alz.com/ltte
